# Ultrasound-based predictive indicators for treatment outcomes in pediatric vesicoureteral reflux

**DOI:** 10.1007/s00595-024-02833-x

**Published:** 2024-04-16

**Authors:** Mehmet Öztürk, Haluk Şen, Feyza Yılmaz, Ömer Bayrak, Gürdal Demirci, Muharrem Baturu, M. Sakıp Erturhan, İlker Seçkiner

**Affiliations:** 1 Department of Urology, Gaziantep 25 Aralık State Hospital, Gaziantep, Turkey; 2 School of Medicine, Department of Urology, University of Gaziantep, 27410 Gaziantep, Turkey; 3 School of Medicine, Department of Radiology, University of Gaziantep, Gaziantep, Turkey

**Keywords:** Vesicoureteral reflux, Submucosal tunnel, Endoscopic injection therapy, Detrusor-to-ureteral orifice distance, Distal ureteral diameter

## Abstract

**Purpose:**

To evaluate the effectiveness of preoperative ultrasound (US) measurements in predicting pediatric vesicoureteral reflux (VUR) treatment outcomes.

**Methods:**

This prospective study enrolled 35 patients (53 renal units) aged 1–16 years who underwent subureteric injection therapy for primary VUR between July 2020 and June 2022. Preoperative ultrasound examinations measured the bladder wall thickness at the ureteral orifice, ureteral submucosal tunnel length, distal ureteral diameter, patient demographics, VUR grade, presenting complaints, bladder–bowel dysfunction, and renal scarring, and the impact of these variables on treatment success was analyzed.

**Results:**

Among the patients, 91.4% were female, with a mean age of 6.83 ± 3.84 years. A comparison between the treatment success and failure groups revealed no significant differences in the age, sex, VUR grade, laterality, bilaterality, presenting complaints, bladder–bowel dysfunction, bladder wall thickness, or distal ureteral diameter (*p* > 0.05). However, renal scarring occurred in 16 (38.1%) patients in the treatment success group and 10 (90.9%) in the treatment failure group (*p* = 0.002). The treatment failure group had shorter detrusor-to-ureteral orifice distances and smaller detrusor-ureteral orifice distance-to-distal ureteral diameter (D/U) ratios than that of the success group (*p* = 0.004 and *p* = 0.006, respectively). Patients with a detrusor-to-ureteral orifice distance < 7.4 mm had an 81.82% likelihood of treatment failure.

**Conclusion:**

Ultrasound measurements of the detrusor-to-ureteral orifice distance and D/U ratio proved reliable in predicting the success of endoscopic subureteric injection therapy for VUR.

## Introduction

Vesicoureteral reflux (VUR) is a common condition in children with a reported prevalence of 1%. However, it is difficult to determine this rate precisely given the varied risk factors in this population. Previous studies have reported a VUR rate exceeding 30% in those with tract infections, and this rate decreases with age [1].

Several factors prevent the development of VUR, among which the anatomical structure of the ureterovesical junction is the most fundamental. The ureter has two fixation points that support it internally and externally [2], and a segment of the ureter between these two support points lying beneath the bladder mucosa creates a tunnel. The length of this subureteric tunnel is five times the diameter of the ureteral orifice and serves as an anti-reflux mechanism [3]. VUR occurs in the presence of a short intramural ureter when the anti-reflux mechanism is disrupted.

Treatment of VUR requires consideration of multiple factors, such as the patient’s age, reflux grade, presence of renal scarring, recurrent urinary tract infections, and bladder dysfunction. Treatment options include conservative approaches, antibiotic prophylaxis, and bladder training, although curative surgical methods, such as endoscopic subureteric injection therapy and ureteroneocystotomy (UNC), are also available.

The present study investigated the values of submucosal tunnel length, distal ureteral diameter, bladder wall thickness, and detrusor-ureteral orifice distance-to-distal ureteral diameter ratio (D/U ratio) measured using preoperative ultrasonography (US) to predict the success of subureteric injection therapy in patients with VUR.

## Materials and methods

The pre- and postoperative data of 35 patients (53 renal units) who underwent subureteric injection therapy for primary VUR between July 2020 and June 2022 were evaluated prospectively.

### Study participants

The parents of the study participants were provided with detailed information about the study and informed consent was obtained. Patients with known secondary reflux or accompanying urinary tract disorders (ureteropelvic junction stenosis, ureterovesical junction stenosis, posterior urethral valve, duplex collecting system, and ureterocele) and those who had previously undergone subureteric injection therapy or ureteroneocystostomy due to VUR were excluded from the study.

Voiding cystourethrography (VCUG), used for the diagnosis and assessment of postoperative success, was performed by an experienced urologist, and the results were interpreted by an experienced radiologist. The International Reflux Study Group (IRSG) 1985 classification was used to determine the VUR grade [4]. The patient age at presentation, sex, presentation data, presenting complaints (febrile urinary tract infection, family history, antenatal hydronephrosis, and abdominal pain), bladder and bowel dysfunction (constipation, urgency, incontinence, and nocturnal enuresis), dimercaptosuccinic acid (DMSA) scan findings, reflux grade, and reflux side were recorded. The eligibility criteria for subureteric injection therapy were recurrent episodes of urinary tract infections during follow-up despite conservative therapy, increased renal scarring on follow-up DMSA scans, non-compliance with conservative therapy, and the presence of indications for surgery, considering the age, sex, and VUR grade.

**Fig. 1:**
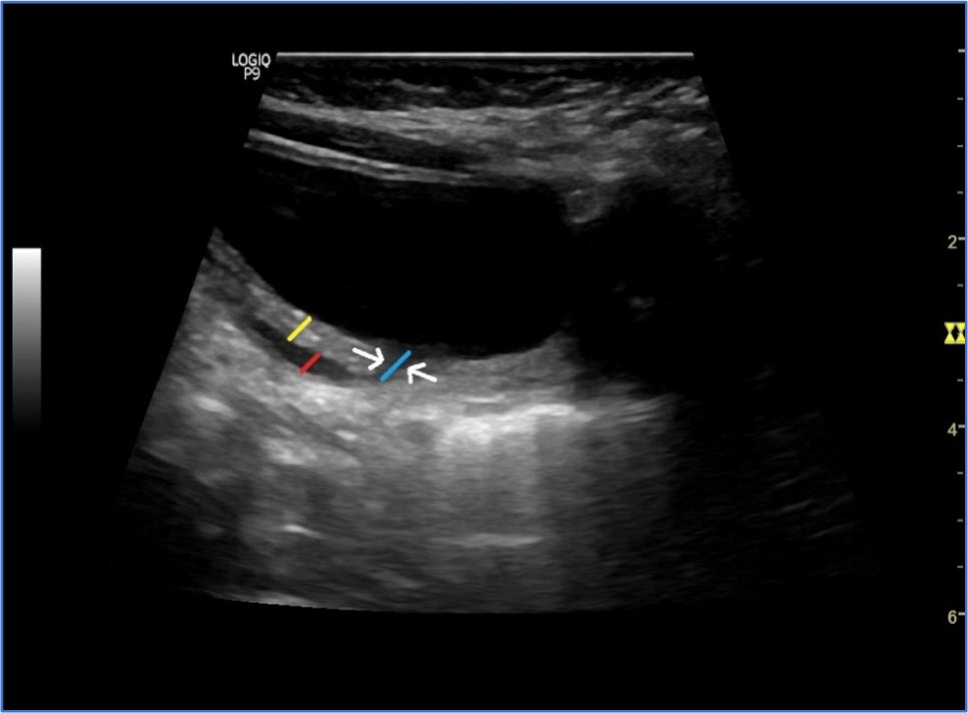
Ultrasonographic measurement. Yellow line: bladder wall thickness. Red line: distal ureteral diameter. Blue line: detrusor-ureteral orifice distance; the white arrow indicates the ureter inside the detrusor

The patients were subjected to a preoperative urinary US examination performed by a single radiologist experienced in the field. The bladder was catheterized and filled according to the bladder capacity formula ([age + 2] × 30) to ensure accurate results before the US examination. In the US examination, the bladder wall thickness at the ureteral orifice, ureteral submucosal tunnel length, and distal ureteral diameter were measured in millimeters (mm), and the detrusor-ureteral orifice distance-to-distal ureteral diameter ratio (D/U ratio) was calculated (Fig. 1).

All patients were discharged on postoperative day 1 with antibiotic prophylaxis. Thereafter, all patients returned 2 weeks later for US control and to undergo urinary dilation, and control VCUG was performed at postoperative month 6 in accordance with the standard follow-up protocol of the clinic. A decline in reflux grade was regarded as a treatment success, and the factors contributing to treatment success were investigated.

### Surgical technique

All surgical procedures were performed by a single urologist using the STING technique. Subureteric injection was performed using a 3.4-Fr cystoscopy injection needle (Cook Medical, Limerick, Ireland). Dextranomer/hyaluronic acid (Dexell® VUR; Istem Medical, Ankara, Türkiye) was used as the injection material in all patients. A urethral catheter appropriate for urethral length was inserted in all patients after the procedure. All patients were discharged on postoperative day 1 after removal of the urinary catheter.

### Radiological Examinations

The ultrasonographic data collected and saved by LOGIQ P9 (General Electrics; GE Medical Systems Information Technologies GmbH, Freiburg, Germany) included images and values obtained through a linear transducer (9C4) probe with a bandwidth of 6 MHz. The images were transferred to a Microsoft-based computer environment via a USB device. US was performed by a different radiologist (Fig. 1).

### Statistical analyses

The Shapiro–Wilk test was used to assess the normality of data distribution, and the Mann–Whitney *U* test was used to compare non-normally distributed variables between the two groups. The relationships between categorical variables were analyzed using the Chi-squared test. A receiver operating characteristic curve was used to determine the cutoff points for the numerical variables. IBM SPSS Statistics (version 22.0; IBM Corp., Armonk, NY, USA) was used for the statistical analysis, and a p-value of < 0.05 was considered statistically significant.

## Results

Of the 35 patients (53 renal units) who underwent subureteric injection therapy due to primary VUR, 32 (91.4%) patients were female and 3 (8.6%) were male, with a mean age of 6.83 ± 3.84 years (treatment failure group: 5.27 ± 3.64 years old; treatment success group: 7.05 ± 4.16 years old; *p* = 0.147) (Tables 1 and 2).

**Table 1:** Demographic and clinical data

	Mean ± SD	Median (min–max)
Age (years)	6.83 ± 3.84	7 (1–16)
	*n*	%
Sex		
Female	32	91.4
Male	3	8.6
Laterality		
Right	6	17.1
Left	11	31.4
Bilateral	18	51.4
Right grade		
1	0	0
2	2	3.9
3	9	16.9
4	6	11.3
5	7	13.2
Left grade		
1	2	3.9
2	3	5.6
3	7	13.2
4	12	22.6
5	5	9.4

*min* minimum, *max* maximum, *n* number, *SD* standard deviation

**Table 2:** A comparison of the baseline characteristics between the groups

		Endoscopic injection successful		*p*
		Yes	No	
Laterality *n*, (%)	Right	19 (45.2%)	5 (45.5%)	0.990
	Left	23 (54.8%)	6 (54.5%)	
Grade *n*, (%)	1	2 (4.8%)	0	0.891
	2	4 (9.5%)	1 (9.1%)	
	3	13 (31%)	3(27.3%)	
	4	14 (33.3%)	4 (36.4%)	
	5	9 (21.4%)	3 (27.3%)	
Bladder–bowel dysfunction *n*, (%)	Constipation	1 (2.4%)	0	0.621
	Incontinence	10 (23.8%)	5 (45.5%)	
	Urgency	1 (2.4%)	0	
	Nocturnal enuresis	6 (14.3%)	1 (9.1%)	
	None	24 (57.1%)	5 (45.5%)	
Presenting complaints *n*, (%)	Antenatal hydronephrosis	5 (11.9%)	4 (36.4%)	0.208
	Febrile urinary tract infection	26 (61.9%)	6 (54.5%)	
	Hydronephrosis + urinary tract infection	4 (9.5%)	1 (9.1%)	
	Family history + febrile urinary tract infection	4 (9.5%)	–	
	Abdominal pain	3 (971%)	–	
Bilaterality *n*, (%)	Unilateral	15 (50%)	2 (40%)	0.678
	Bilateral	15^a^ (50%)	3 (60%)	
Renal scar *n*, (%)	Yes	16 (38.1%)	10 (90.9%)	0.002*
	No	26 (61.9%)	1 (9.1%)	
Age (mean ± sd)		5.27 ± 3.64	7.05 ± 4.16	0.142

^*^p < 0.05 is statistically significant

*n*: number of patients, *SD* standard deviation

A comparison between the groups with regard to the laterality of the reflux, grade, presence of bladder–bowel dysfunction, and presenting complaints revealed no significant differences between the groups. VUR was unilateral in 17 patients (48.6%) and bilateral in 18 patients (51.4%); when these 18 patients were evaluated in terms of treatment success, VUR persisted bilaterally in 3 patients (16.7%) and improved bilaterally in 12 patients (66.6%) and unilaterally in 3 patients (16.7%). The overall success rate in patients with bilateral VUR was 83.3%. The success rate of subureteric injection therapy in 17 patients (48.6%) with unilateral VUR was 88.2%, with no significant difference in treatment success in terms of bilaterality (*P* = 0.678) (Table 2).

An assessment of renal scarring based on preoperative DMSA scans revealed renal scarring in 10 (90.9%) patients in the treatment failure group and 16 (38.1%) patients in the treatment success group. There was a significant difference in the presence of renal scarring between the treatment success and treatment failure groups (*P* = 0.002) (Table 2).

**Fig. 2:**
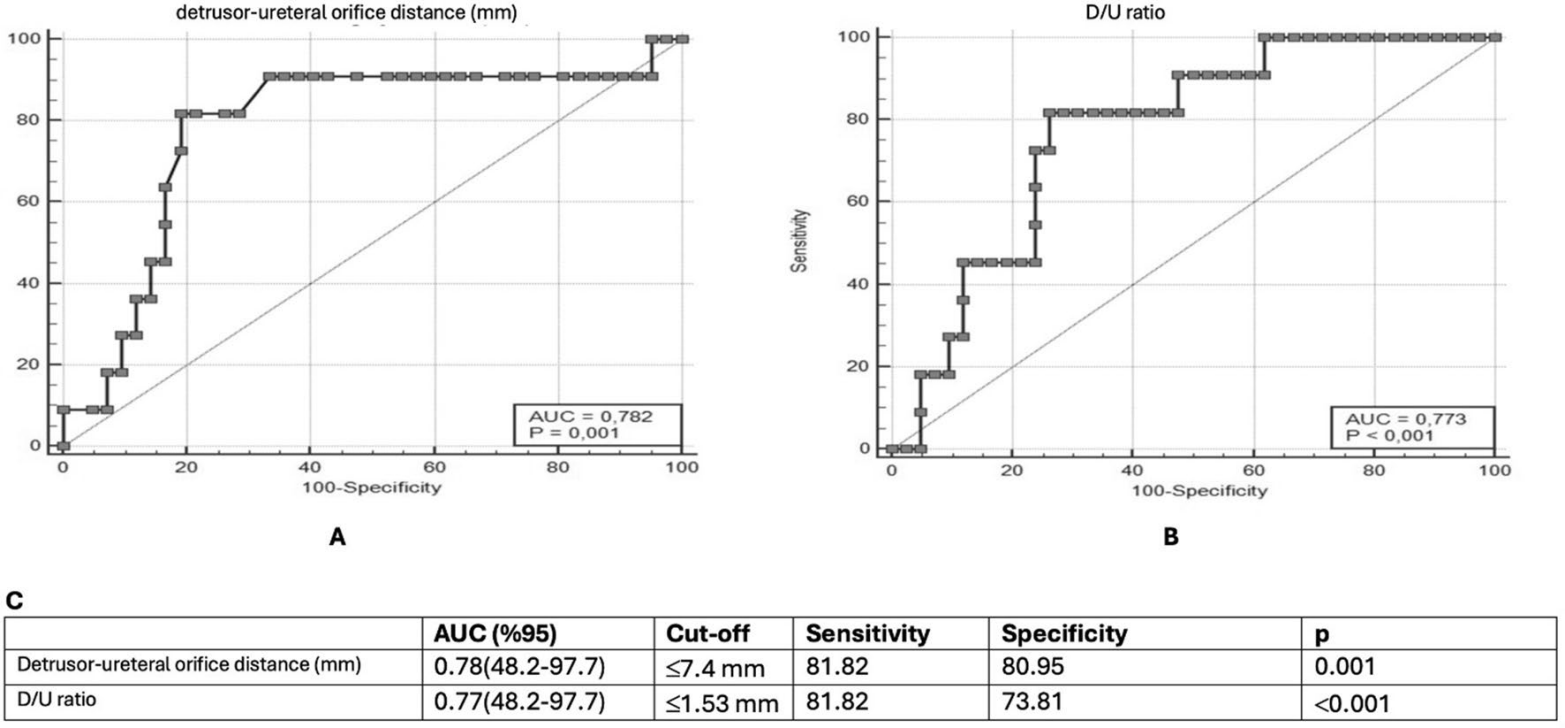
Results of an ROC curve analysis of treatment success according to ultrasound-based factors. **a** An ROC curve analysis of the detrusor-ureteral orifice distance for estimating endoscopic injection success. **b** An ROC curve analysis of the D/U ratio for estimating endoscopic injection success. **c** Results. *D/U* ratio detrusor-ureteral orifice distance-to-distal ureteral diameter ratio, *ROC* receiver operating characteristic, *AUC* area under the curve, *CI* confidence interval

Bladder wall thickness and distal ureteral diameter measured by preoperative US had no association with the treatment success (*p* = 0.163 and *p* = 0.307, respectively) (Table 3). In the treatment failure group, the mean submucosal tunnel length was 7.32 ± 3.15 mm, while in the treatment success group, the mean submucosal tunnel length was 10.64 ± 3.78 mm (*p* = 0.004). The mean D/U ratio of 1.37 ± 0.45 in the treatment failure group was significantly lower than that in the treatment success group (2.12 ± 0.83) (p = 0.006). The submucosal tunnel length and D/U ratio were identified as significant parameters for predicting surgical success (Table 3) (Fig. 2). The likelihood of treatment failure was 81.82% [95 confidence interval (CI) 48.2%–97.7%] in patients with a detrusor-ureteral orifice distance of < 7.4 mm and a detrusor-ureteral orifice distance-to-ureteral diameter ratio of < 1.53 mm (Fig. 2).

**Table 3:** A comparison of successful and unsuccessful groups according to preoperative ultrasonography measurements

	Success of endoscopic injection				*p*
	No (*n* = 11)		Yes (*n* = 42)		
	Mean ± SD	Median [%25–%75]	Mean ± SD	Median [%25–%75]	
Bladder wall thickness (mm)	3.74 ± 0.96	4 [3.1–4.3]	3.44 ± 0.86	3.5 [2.8–4]	0.163
Detrusor-ureteral orifice distance (mm)	7.32 ± 3.15	6.8 [5.9–7.4]	10.64 ± 3.78	10.4 [7.7–13.5]	0.004*
Distal ureteral diameter (mm)	5.35 ± 0.87	5.2 [4.8–6.1]	5.48 ± 3.05	4.95 [4.2–5.7]	0.307
D/U ratio	1.37 ± 0.45	1.36[1.04–1.54]	2.12 ± 0.83	2.07[1.46–2.7]	0.006*

^*^p < 0.05 is statistically significant

D/U ratio: detrusor-ureteral orifice distance-to-distal ureteral diameter ratio, mm: millimeter

## Discussion

The VUR can recover without producing any symptoms but can also result in end-stage kidney disease associated with hypertension and nephropathy. Therefore, it is crucial to prevent or reduce the risk of nephropathy caused by reflux, recognize at-risk patients early, and provide appropriate treatment to those requiring follow-up and treatment. Treatment planning should consider factors such as the sex, reflux grade, type of presentation, presence of lower urinary tract dysfunction, presence of bladder and bowel dysfunction, presence of renal scarring, split renal functions, and compliance of both the parents and the child with therapy [5].

The surgical methods used to treat VUR rely on correcting disturbances in the anti-reflux mechanism. There is no accurate sphincter mechanism between the ureter and bladder that prevents the backflow of urine, as the reflux of urine is prevented by the closure of the intramural ureter by the detrusor muscle with a flap-valve mechanism, making the intramural length of the ureteral orifice an important parameter [6]. Most surgical methods described for treatment are based on the principle that the submucosal tunnel created with sufficient length is closed because of the increasing pressure during the filling and emptying of the bladder. Ideally, the length of the submucosal tunnel should be five times the diameter of the ureter, as proposed by Paquin [3]. In subureteric injection therapy, no change is made in the length of the submucosal tunnel. The injection method provides posterior support to the intramural ureter and point attachment of the ureterovesical junction to the bladder trigone, thereby preventing urine reflux by narrowing, particularly the distal ureter [7].

Endoscopic subureteric injection therapy has become the preferred approach in recent years for the treatment of VUR, even in the presence of high-grade reflux [8]. In the case of a failed first injection attempt, the success rate of the second injection was reportedly 68.3%, and the success rate of the third injection was 34% [9]. Therefore, it is important to predict the success rate of injection therapy accurately. Patients deemed to have a low chance of resolution with injection therapy, even if they have low-grade reflux, may be offered open surgery as first-line therapy, which has a better success rate. The present study therefore investigated the factors that might affect the success of subureteric injection therapy.

Mendez et al. investigated the factors affecting the success of endoscopic injection therapy in 90 children with grade 3–4 reflux. They identified the degree of ureteral dilation as a factor affecting treatment success [10]. In the same study, bilateral reflux was identified as another factor affecting treatment success, with a reported failure rate of 28% in unilateral reflux children and 47% in bilateral reflux (*p* = 0.046). In their study, the degree of ureteral dilation was evaluated using VCUG, whereas US was used in the present study to evaluate the distal ureteral diameter. Similar to the study by Mendez et al., the present study identified a larger distal ureteral diameter in the treatment failure group than that in the treatment success group. However, the difference between these two outcome groups was not statistically significant. The success rate of the injection therapy was lower in patients with bilateral reflux than that of patients with unilateral reflux, although the difference was not statistically significant. The finding of bilateral reflux in 75 patients in the treatment failure group is also consistent with previous studies.

The distal diameter ratio (UDR), calculated by dividing the distance between the L1-L3 vertebra by the distal ureteral diameter at the level of the iliac, has recently emerged as a popular approach to predicting the success of injection therapy, and many studies have been conducted on this subject [11]. In a retrospective study of 70 refluxing children conducted by Helmy et al., the mean distal ureteral diameter was recorded as 5.5 mm, and the mean UDR was 0.38; the authors reported that the UDR was an objective tool for predicting the success of injection therapy for VUR [12]. In another retrospective study involving 79 children, Cooper et al. reported that radiological images of the distal ureter were superior to lower urinary tract imaging in delineating the ureterovesical junction anatomy. They reported that the UDR was higher in patients with high-grade reflux and in patients with treatment failure than that in others and stated that it could be used alone as a better predictor of treatment success than the reflux grade (*p* < 0.0001) [11].

In a study investigating the factors affecting the success of endoscopic injection therapy, Leung et al. identified the preoperative reflux grade and presence of renal scarring as predictive factors in univariate analyses [13]. However, the presence of renal scarring was identified as the only predictor of treatment success in a multivariate analysis. In another study assessing the success of injection therapy, Zambaiti et al. evaluated the correlation between treatment success and the height of the mound produced by bulking the injection material measured by US. They reported that a high mound could prevent reflux following injection therapy, while a mound of 9.8 mm on average could serve as an indicator of reflux resolution [14]. In fact, their study might show that the first postoperative follow-up was performed with US and then only selected patients underwent further VCUG examinations rather than trying to predict treatment success preoperatively.

In a retrospective study of 200 refluxing children conducted by Baydilli et al., the likelihood of treatment failure was 4.068-fold higher in those with VUR in the early phase of bladder filling, 3.076-fold higher in those with a UDR > 0.24, 2.666-fold higher in those with renal scarring on DMSA scans, 2.493-fold higher in those with bladder and bowel dysfunction, 2.341-fold higher in those with febrile urinary tract infection, and 2.745-fold higher in those with delayed contrast agent drainage of the upper urinary tract. A model considering all parameters related to VCUG was better able to predict the outcomes of endoscopic injection therapy than the reflux grade [15]. The authors further highlighted that such predictive parameters could help pediatric urologists select the best candidates for endoscopic injection therapy. The present study found no marked effect of bladder or bowel dysfunction, presenting complaints, or reflux grade on the treatment success.

In patients experiencing reflux, the risk of febrile urinary tract infection increases in parallel with reflux grade [16]. In a study by Payza et al., the UDR was higher in patients with recurrent febrile urinary tract infection as the presenting complaint and in those with a positive family history of VUR than that in those with none of these factors, although the difference was not statistically significant (*p* = 0.372). In the same study, a relationship was suggested between the UDR and the clinical course of reflux, consistent with the results of previous studies. Furthermore, the UDR was significantly higher in patients with moderate-to-severe renal scarring identified on the DMSA scan than that in patients with mild and non-renal scarring (*p* = 0.001). Similar to our findings, the authors reported no significant correlation between the presence of bladder and bowel dysfunction and UDR [17].

Many studies have demonstrated the value of the distal ureteral diameter measured using VCUG and the UDR in predicting the success of endoscopic injection therapy. To our knowledge, no study has evaluated the distal ureteral diameter, detrusor-to-entrance ureteral orifice distance, or D/U ratio measured by US. The present study, therefore, makes a significant contribution to the literature as the first study of this issue and in its reporting of results that are different from those of earlier studies. The present study found no relationship between the success of endoscopic injection therapy and the distal ureteral diameter, presence of bladder or bowel dysfunction, presenting complaints, or patient’s age and sex. Unlike previous studies, no relationship with distal ureteral diameter was identified, possibly due to the small sample size. However, the study findings suggest that the length of the submucosal tunnel is a protective factor against reflux, while the D/U ratio, together with renal scarring, is an independent predictor of success in endoscopic injection therapy, and a tunnel length of < 7.4 mm is associated with an 81.82% likelihood of failure.

Several limitations associated with the present study warrant mention, including the small sample size, concentration of radiological findings only, and short-term follow-up. The current results need to be confirmed in prospective randomized studies with larger patient series and long-term follow-up.

## Conclusion

We believe that the prediction of treatment success in endoscopic injection therapy is crucial for selecting appropriate patients for injection therapy. In the current study, the tunnel length and D/U ratio measurements obtained by US examination indicated a likelihood of treatment failure and could be used as negative predictive values. Large-scale comprehensive studies are required to confirm the findings of this study.

## Data Availability

The data are freely available upon reasonable request from the corresponding author.
